# Striking Concerns: Reduced Protection in Older Kendo Helmets

**DOI:** 10.7759/cureus.102889

**Published:** 2026-02-03

**Authors:** Ellison Wong, Harrison G Chu, Alexis Leo, Gary Chu

**Affiliations:** 1 Medicine, California Northstate University College of Medicine, Elk Grove, USA; 2 Internal Medicine, California Northstate University College of Medicine, Elk Grove, USA

**Keywords:** concussion prevention, equipment safety, helmet design, helmets, helmet safety, kendo, martial arts, old equipment, protective gear, safety standards

## Abstract

Background: Protective headgear in many sports, such as cycling and skiing, is subject to clear recommendations regarding replacement intervals due to material degradation over time. In contrast, kendo helmets (men) lack evidence-based guidance on service life, despite repeated exposure to high-energy impacts during regular practice. In our prior study, we noted that new kendo helmets of different stitch patterns absorb kinetic energies differently.

Objective: To evaluate the effectiveness of older kendo helmets compared to new helmets in mitigating impact forces that could lead to sports-related concussions in kendo.

Methods: We collected data from seven kendo practitioners and collected over 1,900 strikes using a bamboo sword (shinai) on the same sensor-equipped mannequin from our prior study on five helmets over five years old with a 6-mm stitch pattern, tested under controlled conditions simulating realistic impacts encountered in kendo practice, using the same protocol as our prior study.

Results: Older helmets consistently demonstrated lower energy absorption, exhibiting statistically significant higher mean g-forces, with a 95% confidence interval (CI), compared to new helmets with different stitch patterns (2 mm, 6 mm, and 9 mm) from our prior study (p < 0.001). Older helmets recorded an average of 17.9 g, compared with prior study values of 13.5 g for 9-mm helmets, 13.9 g for 6-mm helmets, and 14.7 g for 2-mm helmets (p < 0.001). This corresponds to respective decreases in energy absorption of 32.8%, 29.0%, and 22.0%. A comparison of the worst-performing old helmet (Old Helmet 3) with the best-performing new helmet (9-mm stitching) showed a reduction of 36.7% (18.5 g vs. 13.5 g).

Conclusions: These findings indicate that older kendo helmets provide substantially reduced protection (22%-36.7%) compared to new helmets. Given the frequency of head impacts in kendo and the reduced energy absorption of older helmets, continued use of aged equipment may pose a safety risk. The results highlight the need for evidence-based recommendations regarding helmet replacement intervals in kendo to enhance practitioner safety.

## Introduction

Kendo, often referred to as Japanese-style fencing, is a traditional martial art that has grown into a modern international sport. Today, it is practiced in over 60 countries with several million practitioners [1]. A typical kendo match involves two participants who attempt to score points by landing strikes with a bamboo sword known as a shinai. The protective gear (bogu) consists of a helmet (men), chestplate (do), and thigh padding (tare). Points are awarded for accurate strikes to the head, throat, wrists, or torso [1]. 

Although it is considered a relatively safe contact sport, injuries do occur. Most reported injuries are soft-tissue in nature, with head or concussive injuries representing a small proportion of injuries [2]. Soft tissue injuries will generally resolve with rest; however, brain trauma leads to the risk of chronic traumatic encephalopathy (CTE), which results in chronic cognitive, motor, mood, and behavioral impairment [3]. In a study on subjects with CTE, 16% had an absent history of concussions, highlighting the danger of cumulative subconcussive impacts and emphasizing the need to mitigate such impacts in kendo practitioners [4]. Other studies support the idea that repeated subconcussive impacts are sufficient to impair cognition and may result in physical changes in neuroanatomy [4]. Our prior study showed that head strikes on various new kendo helmets generally produce impact forces below those associated with concussion. However, new helmets with different stitch patterns (2 mm, 4 mm, 6 mm, 9 mm, etc.) exhibit differing shock-absorption properties and varying ability to mitigate the force generated by a shinai during a standard head strike in practice [5]. Additionally, subconcussive head traumas may not solely result in insidious neurological defects; a Japanese case report documented a kendo practitioner who developed traumatic brain injury-related epilepsy linked to brain traumas sustained from practicing kendo [6]. 

Unlike other contact sports such as football, hockey, skiing, or cycling, kendo lacks standardized equipment regulations. The International Kendo Federation (FIK) provides some guidelines, mainly regarding the length, weight, and material of the shinai, but offers limited recommendations for the helmets [7]. In contrast, sports like football have extensive regulations governing helmet construction, fit, and replacement. According to standard ND 001 6.1.1 established by the National Operating Committee on Standards for Athletic Equipment (NOCSAE), football helmets must be inspected and pass performance testing every two years [8]. Similarly, in skiing and cycling, the U.S. Consumer Product Safety Commission (CPSC) enforces government-mandated helmet safety standards [9]. It is recommended to replace motorcycle, bike, and ski helmets following any crash where the head hits the ground, as well as visible defects such as cracks in the outer shell, frayed straps, missing pads, or fading of the shell that may indicate ultraviolet (UV) damage [9]. While a third party exists to ensure the quality of kendo bamboo swords, no third-party organization exists to evaluate or certify the protective performance of kendo gear [10]. 

Similarly, there are no guidelines regarding proper maintenance and replacement timelines of kendo helmets. Kendo helmets are primarily made of leather, which is known to stiffen with aging, potentially impairing their compressive properties essential for attenuating impacts [11,12]. Natural fibers present within kendo helmets are subject to structural damage from chronic exposure to elevated temperatures, compromising the flexibility essential for absorbing impacts [13]. Studies on environmental effects on helmets for contact sports such as football similarly find that heat and humidity reduce absorptive ability [14]. Kendo helmets, which are made of these materials known to decrease in elasticity and compressibility when subjected to environmental factors, will likely show a decrease in protective ability with aging. 

A force benchmark of 19 g has been correlated with clinical brain injury in comparative soccer-impact studies [15,16]. It remains to be determined whether impacts sustained while wearing kendo helmets approach this threshold.

In our prior study, we compared materials and padding in newer helmet models and found that wider 9-mm stitching and the addition of polyurethane-based padding provide the greatest protection against concussion from head strikes [5]. However, it remains unclear whether the protective performance of kendo helmets degrades over time, and there are currently no official recommendations from kendo organizations regarding helmet replacement.

Our prior study also suggests that the protective effectiveness of kendo helmets is primarily determined by stitch pattern rather than materials or manufacturer [5]. Building on this finding, this study aims to empirically determine whether the protective ability of kendo helmets decreases with age and repetitive use. Specifically, it examines whether new helmets provide greater protection than helmets that are five years old. Establishing this relationship could help generate a discussion on safety recommendations and replacement guidelines. As kendo continues to grow worldwide, ensuring that practitioners train and compete with adequately protective equipment is essential to the sport’s safety and longevity.

## Materials and methods

Seven kendo practitioners with different experience levels (two beginners, three kyu-level, and two dan-level) participated in this study. Together, they performed over 2,000 standard men strikes (straight downward strikes) on five kendo helmets that had been in use for more than five years.

A third-dan kendo practitioner supervised all testing to ensure safety and consistency and provided guidance so that the strikes reflected normal dojo practice. All strikes followed a standardized procedure, including strike location, stance, and helmet positioning.

All participants met Institutional Review Board requirements: they were between 18 and 60 years old, in good physical health, and not pregnant. The five tested helmets were all older than five years and used the same 6-mm stitching pattern. Standard, commercially available practice-grade shinai were used, with each practitioner using their own shinai throughout the study.

The tested helmets were sourced from three distinct manufacturers and were donated by various kendo practitioners in Japan. Each tested helmet was used for over five years before this study and is referred to as an old helmet. Practitioners did not know which helmet was being tested at the time of each strike to minimize bias and ensure consistent strike force. A total of 1,931 strikes were recorded, in segments of roughly 50 consecutive strikes followed by a break to account for fatigue. This study uses the design protocol specified in our prior study [5]. To allow for performance evaluation of the old helmets examined in our study compared to the new helmets in our prior study, the same mannequin, impact force sensor, and experimental setup used in our prior study were used. Four kendo practitioners returned from the original study, with new volunteers accounting for the remaining data. 

**Figure 1:**
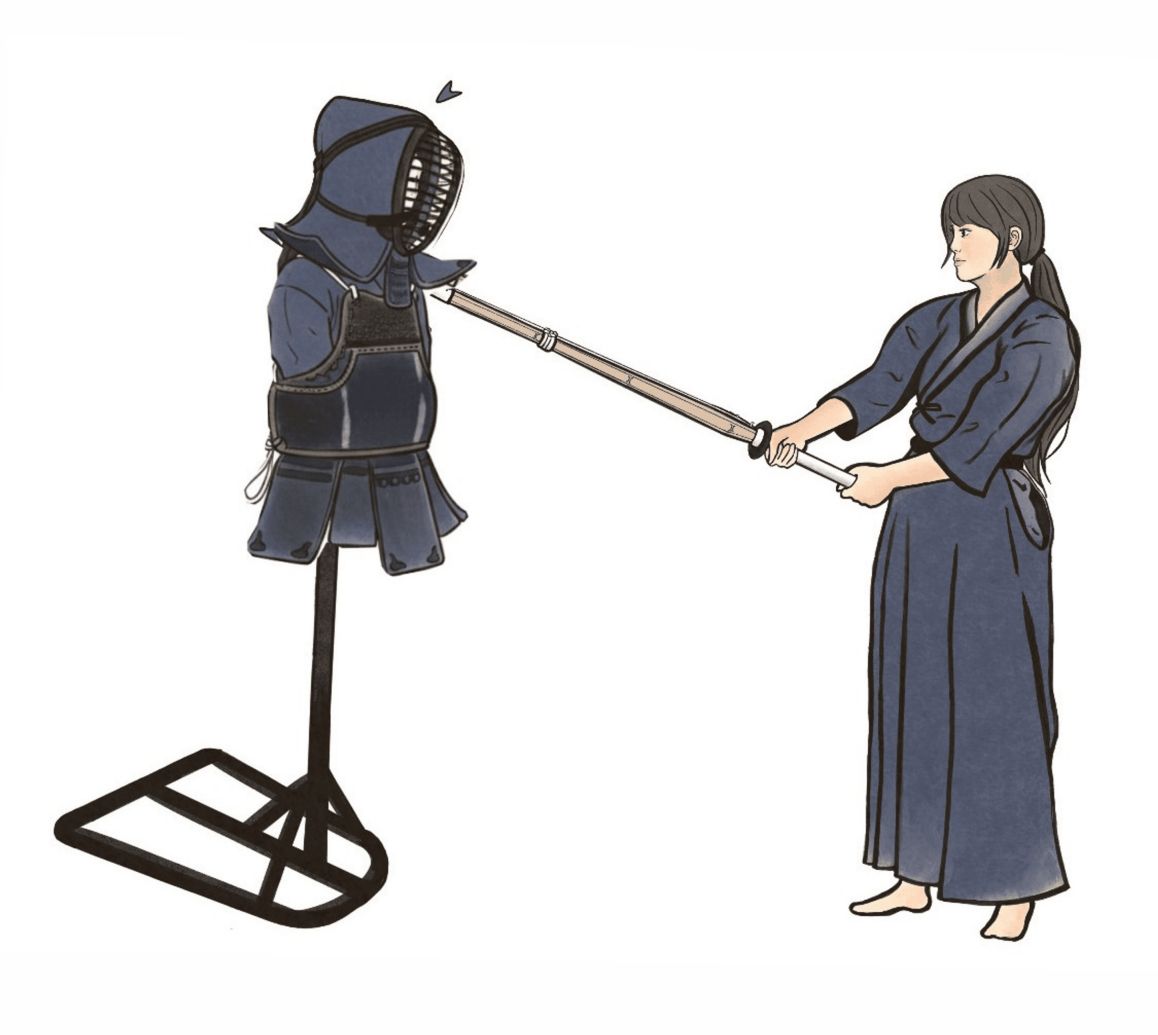
Experimental set-up Kendo practitioner executing a vertical strike with a shinai on a mounted kendo helmet fitted with a linear acceleration sensor. *Identical set-up and equipment utilized from a prior study by Ta et al. [5]. Figure drawn by Megan Hsu.

We mimicked the proportions of a realistic kendo opponent by mounting a mannequin head with anthropomorphic dimensions for a male (57 cm height, 33 cm circumference) on a 175 cm tall post (the average height of a male in the United States) (Figure 1). A force of impact sensor (NETPLAYZ, Gardena, CA, USA) was embedded with silicone in the cranial region of the mannequin, capturing the linear acceleration experienced by the model upon vertical strikes. Standard fastening was used to attach the tested helmets to the mannequin head, ensuring the fit would most accurately represent that of a real practitioner. 

For each helmet, datasets contained at least 350 measured strikes combining contributions from practitioners representing different levels of experience (two beginner, three kyu-level, and two dan-level). The average force upon impact for each tested helmet was compared to commercial off-the-shelf helmets tested in our original study by Ta et al. [5]. A san dan (third-degree black belt) practitioner was present during data collection to ensure safety, and the strikes rendered are similar to a dojo practice. 

The average g-force and its 95% confidence interval (CI) for each old helmet was compared to the average g-force and 95% CI of the best and worst performing new helmets from the original study. Data analysis was performed using Google Sheets (Google LLC, Mountain View, CA, USA) with the XLMiner Analysis add-on (Frontline Systems Inc., Incline Village, NV, USA). Subsequent graph analysis was done through Google Sheets.

## Results

**Figure 2:**
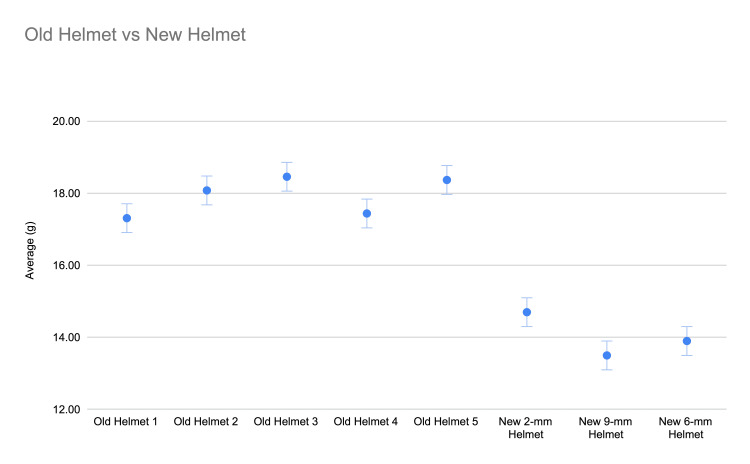
Measured impact differences between old helmets and new helmets The data are represented as mean (center dots) and 95% confidence intervals. Differences between subgroups of only old helmets (F(4, 1926) = 8.23, p < 0.001) and all helmets (F(7, 3170) = 142.76, p< 0.001) analyzed with one-way ANOVA test.

A total of 3,178 strikes were analyzed across all helmet conditions, including 1,931 strikes collected on the aged kendo helmets (Helmets 1-5). Each tested helmet received at least 350 strikes to account for impact variability. Table 1 summarizes the impact data for each helmet condition. The same data is graphically represented in Figure 2 with error bars representing 95% CI’s; none of the intervals for the old helmets overlap with any of the new helmets. This suggests there may be statistically significant performance differences between new and old helmets. Although these differences appear modest in absolute magnitude, the relatively narrow CIs and large sample sizes indicate consistent performance differences among helmet models. The mean linear acceleration for the aged cohort was 17.92 g (95% CI = 0.13 g, SD = 3.67 g), the best-performing old helmet was 17.31 ± 0.33 g (Old Helmet 1), and the worst-performing was 18.46 ± 0.41 g (Old Helmet 3). A one-way ANOVA test determined a significant statistical difference between all helmets in the old subgroup (F(4,1926) = 8.23, p < 0.001). A one-way ANOVA across all helmets included in the study (old and new) also demonstrated statistical significance (F(7,3170) = 142.76, p < 0.001). 

**Table 1:** Measured impact data (g) from old helmets Differences between subgroups of only old helmets (F(4, 1926) = 8.23, p < 0.001) and all helmets (F(7, 3170) = 142.76, p< 0.001) analyzed with one-way ANOVA test. *Data taken from a prior study by Ta et al [5].

	Average g-force (g)	95% Confidence Interval (CI)	Standard Deviation (SD)	Upper 95% CI	Lower 95% CI	Sample Size (n)
Old Helmet 1	17.31	0.33	3.47	17.64	16.98	429
Old Helmet 2	18.08	0.35	3.63	18.43	17.73	404
Old Helmet 3	18.46	0.41	4.02	18.87	18.05	370
Old Helmet 4	17.44	0.34	3.34	17.78	17.44	362
Old Helmet 5	18.37	0.38	3.75	18.75	17.99	366
2-mm New Helmet*	14.70	0.30	3.34	15.00	14.40	410
9-mm New Helmet*	13.50	0.30	2.77	13.80	12.90	422
6-mm New Helmet*	13.90	0.30	3.27	14.20	13.60	415
Average old Helmet	17.92	0.13	3.67	18.05	17.79	3,178

As detailed in Table 1, the standard deviation (SD) for impacts on old helmets ranged from 3.34 g to 4.02 g, indicating that aged helmets not only transmit higher average forces but also exhibit wider impact variation compared to new helmets. The 9-mm new helmet exhibited the lowest mean linear acceleration (13.5 ± 0.3 g) and the lowest SD (2.77) out of the new helmet subgroup (Table 1). 

To quantify performance differences between old and new helmets, the aged helmet data were compared against data established in our prior study of commercial off-the-shelf helmets (2-mm, 6-mm, and 9-mm stitch pitches). These comparisons are shown as % differences in Table 2. The average-aged helmet allowed for 32.7% more linear acceleration than the best-performing new helmet (9-mm New Helmet) (17.92 g vs. 13.5 g; difference = 4.4 g). Compared to the 6-mm New Helmet, the aged helmets performed 28.9% worse (17.92 g vs. 13.9 g; difference = 4.0 g) (Table 2). Compared to the 2-mm New Helmet, the aged helmets performed 19.5% worse (17.92 g vs. 15.0 g; difference = 2.9 g) (Table 2). The largest observed difference was between Old Helmet 3 and the 9-mm New Helmet (36.7% performance reduction; 18.46 g vs. 13.5 g; difference = 5.0 g). As seen in Figure 2, these differences are statistically significant as the CIs between these groups do not cross.

**Table 2:** Measured impact (g) comparison between old (>5 years) and new helmets Represented as percentage (%) difference calculated as (Old-New) / (New) x 100%. Rows 1-3 compare a grouped dataset of all old helmets (Average Old Helmet) to new helmets of different stitching densities (2-mm, 6-mm, 9-mm New Helmet). Row 4 compares the worst-performing aged helmet (Old Helmet 3) to the best-performing new helmet (9-mm New Helmet). Differences between subgroups of only old helmets (F(4, 1926) = 8.23, p < 0.001) and all helmets (F(7, 3170) = 142.76, p< 0.001) analyzed with one-way ANOVA test. *Data taken from a prior study by Ta et al [5].

Average Old Helmet (g)	Sample Size (n)	2-mm New Helmet* (g)	Sample Size* (n)	Difference	% differences
17.92	3,178	15.0	410	2.9	19.5%
Average Old Helmet (g)	Sample Size (n)	6-mm New Helmet* (g)	Sample Size (n)	Difference	% differences
17.92	3,178	13.9	415	4.0	28.9%
Average Old Helmet (g)	Sample Size (n)	9-mm New Helmet (g)	Sample Size (n)	Difference	% differences
17.92	3,178	13.5	422	4.4	32.7%
Old Helmet 3 (g)	Sample Size (n)	9-mm New Helmet (g)	Sample Size (n)	Difference	% differences
18.47	370	13.5	422	5.0	36.7%

## Discussion

We sought to investigate whether older kendo helmets lose some of their protective ability against head strikes in kendo. We compared our data to the 2-mm stitching and 9-mm stitching new helmets from a prior study because these models represented the least and most protective headgear, respectively, among the new commercial off-the-shelf helmets. In our testing, the five old helmets produced significantly greater g-force values than the 2-mm stitching new helmet, the 6-mm stitching new helmet, and the 9-mm stitching new helmet (p < 0.001) when compared to data from that prior study [5]. On average, the heavily used old helmets reacted to impact with a 22.0% higher linear acceleration than the 2-mm stitched new helmet, which was the lowest performance among the six new helmets tested in the prior study. The highest-performing new helmet, featuring 9-mm stitching, exhibited a linear acceleration of 13.5 g, representing a substantial 32.8% reduction in g-force compared with the average of the old helmets. These findings suggest that helmets older than five years may allow greater linear acceleration of the head in comparison to new kendo helmets. 

Several factors may explain the decreased protection by older, heavily used kendo helmets. One is the reduced capacity of materials to absorb impact after repeated smaller strikes. Studies on American football helmets and equestrian helmets have shown decreased ability to attenuate linear acceleration following numerous impacts [17,18]. These helmets differ from kendo helmets in design and materials, but the same general principle of material fatigue may apply. Traditional kendo helmets are constructed primarily from an outer stitched layer with inner padding made of leather and cotton. This was likely the composition of the helmets used in our study, though their exact construction is proprietary.

Sweat and moisture trapped in the helmets promote bacterial growth, which can lead to enzymatic degradation of leather and cotton fibers. As fibers break down, they may lose tensile strength and elasticity. Compression of the padding can also reduce internal air buffering that contributes to energy absorption [19]. Accumulated salt from sweat may additionally stiffen these materials by inserting into the porous collagen matrix [20]. Heat exposure further reduces the elastic and tensile properties of cotton and leather by degrading cellulose and collagen fibers [13]. For these reasons, it is recommended that practitioners wipe down their equipment after every use to minimize moisture retention and store gear in a well-ventilated area away from sunlight [21]. Stitching is also likely to loosen or thin out after cyclic stress, reducing its ability to disperse force [22]. These environmental factors may all be potential causes for the observed reduction in protection against head strikes in the older helmets.

Though brain injuries in kendo are rare, concussions may still occur, as evidenced by a survey of Japanese high school athletes, which observed four kendo practitioners who suffered concussions [23]. Concussions are known to produce neurological symptoms such as problems with memory, attention, concentration, mood, and behavior [24]. In severe cases, sustaining a second concussion before the first has healed may result in second-impact syndrome, a rapidly progressive and life-threatening cerebral swelling [25]. Despite its low incidence rate, the potential for serious and even fatal injury makes protecting against head injuries a critical consideration in the practice of kendo, despite its rare occurrence. A study on concussions in football determined an average peak linear acceleration of 62.4 g and 102.5 g for youth and adults, respectively [26]. The old helmets tested in this study provided sufficient protection against head strikes to fall under the concussion threshold for youth and adults; however, this does not preclude kendo practitioners from suffering brain injuries. Subconcussive forces have been associated with white matter damage and cognitive impairment [4]. This is supported by a study finding that heading in soccer (estimated impact of 19 g only) is associated with these structural and cognitive effects [15,16]. Cumulation of subconcussive brain traumas has also been linked to CTE, which consists of chronic neurological symptoms impairing cognition, motor function, mood, and behavior [4]. Interestingly, none of the new helmets demonstrated average peak accelerations greater than 15 g, while the old helmets tested reported averages around 18 g. Assuming a normal (bell-shaped) distribution, Old Helmet 3 is expected to experience forces above 19 g approximately 44.7% of the time (z = 0.134). In contrast, a new 9 mm helmet is expected to experience forces above 19 g only about 2.3% of the time (z = 1.56) (Table 3). This theoretical disparity seems to suggest that older helmets may offer less protection against head strikes than newer helmets. Lastly, because the same sensor and experimental setup were used as in the prior study of new kendo helmets, the present data likely allow for a meaningful comparison of relative force-reduction performance, even though absolute acceleration values may be affected by measurement uncertainty. We acknowledge that striking variation between practitioners represents an inherent limitation of this cross-sectional design. We attempt to compensate for this by gathering a large amount of strike data from multiple kendo practitioners.

**Table 3:** Estimated proportion of high-magnitude strikes (≥19 g) across helmet conditions Percentages above 19 g were estimated by using the mean g-force and SD values to model a normal distribution for each helmet configuration. Z-scores were then used to approximate the proportion of strikes exceeding the 19 g cutoff. The threshold value (19 g) is based on a comparative study on soccer heading, where repetitive impacts around 19 g correlated with cognitive and neuroanatomical changes [15,16]. *Data taken from a prior study by Ta et al. [5].

	Percentage of Strikes >19 g	Average g	Standard Deviation (SD)
Old Helmet 1	31.3%	17.31	3.47
Old Helmet 2	40.0%	18.08	3.63
Old helmet 3	44.7%	18.46	4.02
Old Helmet 4	32.0%	17.44	3.34
Old Helmet 5	43.3%	18.37	3.75
Average Old Helmet	38.5%	17.92	3.67
2-mm New Helmet*	9.9%	14.70	3.34
6-mm New Helmet*	5.9%	13.90	3.27
9-mm New Helmet*	2.3%	13.50	2.77

Limitations

Though our physical findings of poorer protection in aged helmets should raise concern about the potential for brain injury, no conclusive statements can be made on the medical consequences of using aged helmets without corresponding clinical data. We find that older kendo helmets (five years or older) have reduced protective ability against linear acceleration from vertical head strikes; however, we cannot conclusively correlate this to clinical findings. Our study sought to replicate the vertical strikes conducted in a kendo dojo practice environment, but it does not accurately represent the frequency and intensity of head impacts from shinai strikes in a live match, which limits the generalization of our findings to real kendo practitioners. We did not collect clinically relevant data such as symptoms, concussion diagnosis, neuroimaging, or cognitive testing, preventing us from drawing conclusions regarding the clinical consequences of head impacts in kendo. Building on the original impact data from Ta et al., our findings suggest that kendo helmets aged over five years allow greater impact forces than off-the-shelf models during downward head strikes [5]. This observed increase in force transmission highlights the need for further studies to investigate whether these biomechanical differences translate to measurable increases in clinical injury risk. A longitudinal study on kendo practitioners combining magnetic resonance imaging/diffusion tensor imaging measurements of white matter integrity, neurocognitive testing (e.g., neuro exams, balance assessments), and real-world head-impact data could provide valuable insight into the effects of repeated kendo downward head strikes on brain health. 

Another limitation of this study was the lack of measurement of rotational acceleration. Rotational acceleration causes shear forces within the brain and is closely linked to traumatic brain injuries [27]. Although many helmet-safety experiments focus primarily on linear acceleration, rotational acceleration is also an important predictor of neurological injury [28]. In other combat sports, such as taekwondo, practitioners experience high levels of rotational acceleration even while wearing protective headgear [28]. The vertical strikes we tested are less likely to induce substantial rotational acceleration compared to other legal kendo strikes, such as side strikes to the helmet or thrusts to the throat. Furthermore, drop tests using mannequin heads often underestimate rotational forces because the neck of the test model is too stiff to mimic the motion of a real person. To better capture rotational acceleration, future experiments could use sensors with gyroscopes in a similar setup. The mounted mannequin head used in the experiment does not recreate the force-dissipating properties of active muscular contraction that occur in real head impacts [29]. To address the fixed-mount-head-form limitation of mannequin models, practitioners could use mouthguard-based sensors during real practices and matches to collect data with a more accurate representation of real linear and rotational accelerations experienced by real head strikes in kendo.

The target sensor was not calibrated against another device, which may have led to inaccuracies in the linear acceleration measurements. Nevertheless, the principal trend from this experiment, compared to measurements of the new helmets in a prior study by Ta et al., which utilized the same target sensor, allowed for the trend to be valid [4].

We aimed to represent a live dojo practice environment, characterized by variable impacts from human inconsistency. To compensate for the inherent variability, we gathered a large dataset (>350 strikes for each helmet) to generate tighter confidence intervals and more stable mean estimates. This approach aligns with established strategies in sports impact research; for instance, a study on high school football head impacts employed high-volume data collection to address inherent statistical variability and ensure meaningful results [30]. Keeping in mind the differences in striking force between skill levels established by Ta et al., we recruited a diverse cohort (two beginners, three kyu-level, and three dan-level) to reflect the mixed skill levels found in a live-dojo environment [5]. Supervision by a san dan (third-degree black belt) practitioner promoted consistent stance, strike location, and striking technique between volunteers. Additionally, we collected a large sample size (>350 strikes per helmet) to mitigate the influence of individual strike variability through statistical averaging. Four of the participants returned from the original study by Ta et al., and the remaining data were supplemented by new participants. We acknowledge that striking variation between practitioners represents an inherent limitation of this cross-sectional design, which should be considered when interpreting the differences in new versus aged helmets. 

Another inherent limitation of mannequin-based testing is the failure to reflect natural variability in head shape. Helmet fit differs based on head shape, and a poor fit will cause the head to move more independently of the helmet, increasing risk for concussion [31]. The old helmets were previously worn by kendo practitioners, unlike the newer kendo helmets, and it is possible that the older helmets had greater material plasticity due to being broken in. Despite any potential advantages of a more conformed fit, the older helmets still performed significantly worse than both new helmets.

With the exact usage history and storage conditions of the old helmets unknown, the findings are not generalizable to all kendo helmets older than five years. Further testing with a greater number of older helmets and those with known storage conditions with controlled temperature and humidity may allow for more generalizable findings. The aged Kendo helmets were donated by a Kendo practitioner in Japan and were manufactured by three different brands. We acknowledge the limited scope of the helmets tested in our study, which does not represent all brands, manufacturing methods, material makes, and usage conditions. All helmets tested in our study employed a 6-mm stitching pattern, the most common configuration among beginner- to intermediate-level kendo practitioners. This market context was obtained via personal communication with Nathan Gallagher and Jeff Chen, operators of Kendo armor retail businesses in the United States. These individuals were contacted solely for industry knowledge and were not affiliated with the study; no helmets analyzed in this study were supplied by either individual or their respective businesses. While our findings may apply to a typical entry-level to mid-range kendo helmet with typical usage in US dojos, extrapolation of these findings to helmets with different material makes, brands, manufacturing methods, and usage conditions should be cautiously approached and warrants a broader investigation. 

Furthermore, different testers using different shinai may affect the outcome of the study. We limited this variable because each tester uses their own, the same shinai, during the entire test.

Lastly, kendo is deeply rooted in tradition, and practitioners are often reluctant to adopt changes, even when new safety technologies become available, such as eye shields designed to protect against splinters flying off the shinai during a head strike [32]. As a result, behaviors may not change even when data show that older helmets provide less head protection than newer designs.

## Conclusions

Our findings demonstrate a significant reduction (22%-33%) in the ability of older kendo helmets (>5 years of use) to attenuate linear acceleration compared with new helmets, indicating an age-related degradation of protective performance. Notably, older helmets tested produced average peak accelerations reaching 19 g, a threshold suggested in the literature to be associated with microstructural brain changes and cognitive anomaly, comparable to the head acceleration experienced during soccer heading. In contrast, none of the new helmets evaluated exhibited average accelerations greater than 15 g. Given the frequency of head strikes inherent to kendo practice, the continued use of older helmets that approach this threshold suggests a possible safety concern. Although this study did not directly assess neurological outcomes, minimizing exposure to elevated head acceleration remains a prudent and necessary risk-mitigation strategy. Based on these findings, the continued use of kendo helmets beyond approximately five years is discouraged, and regular inspection and timely replacement of protective equipment are strongly advised to enhance practitioner safety.
